# E-commerce adoption and farmers’ pesticide reduction behavior: implications for food system security

**DOI:** 10.3389/fnut.2025.1665628

**Published:** 2025-10-14

**Authors:** Hailan Qiu, Wenyi Tang, Hanyun Deng, Lun Hu, Jiawei Wang, Biao Sheng, Wenmei Liao

**Affiliations:** ^1^School of Economics and Management, Jiangxi Agricultural University, Nanchang, China; ^2^Digital Faculty of Economics, Jiangxi Open University, Nanchang, China; ^3^School of Systems Science and Engineering, Sun Yat-sen University, Guangzhou, China; ^4^Research Center for the Three Rural Issues, Jiangxi Agricultural University, Nanchang, China

**Keywords:** e-commerce adoption, pesticide reduction, farmers, food safety, policy optimization

## Abstract

**Introduction:**

Reducing pesticide is an important measure to ensure the quality and safety of agricultural products and promote the development of organic agriculture. E-commerce effectively promotes the reduction of pesticide use by guiding consumers’ demand for agricultural products with low pesticide residues.

**Methods:**

Taking 3531 farmers in China as a sample, this paper analyzes the impact and mechanism of e-commerce adoption (ECA) on farmers’ pesticide reduction behavior (FPRB) by using the binary Logit model and mediation effect model, and investigates the heterogeneous impact of ECA on FPRB. On this basis, the differences in the effects of different e-commerce scales, modes and pesticide application methods are further discussed.

**Results and discussion:**

The research findings demonstrate that ECA effectively promote FPRB, with agricultural income expectations, food safety perception, and adoption of agricultural machinery socialization services serving as positive mediating factors. Heterogeneity analysis reveals that the impact of ECA on FPRB varies across individual levels, family levels, and regional levels. Further exploration indicates that large-scale e-commerce platforms and socialized e-commerce models exhibit more significant effects in FPRB, while ECA better facilitate farmers’ adoption of mechanized pesticide application methods. By quantifying the impact of ECA on FPRB, this study demonstrates the effective role of e-commerce in expanding the three core benefits of ecological protection, product safety and sustainable development of organic agriculture, and provides important reference and empirical enlightenment for the development of e-commerce and the green transformation of agriculture in developing countries.

## 1 Introduction

As an indispensable means of production in modern agricultural industry, pesticides play an important role in preventing and controlling crop diseases and pests and ensuring national food security (1–4). Moreover, the increasing global population has led to an increasing demand for food and the increasing use of pesticides to ensure agricultural productivity and efficiency (5). However, excessive use of pesticides has also led to a series of ecological problems. The massive loss of pesticides will pollute soil and water, destroy ecological balance and affect the sustainable development of agriculture (6–8), destroy ecological balance and affect the sustainable development of agriculture (6). Pesticide residues not only affect the quality and safety of agricultural products, but also may pose a serious threat to human health (9). As a major agricultural country in the world, in recent years, China has been devoted to exploring new ways to reduce pesticide consumption, such as replacing chemical pesticides with biological pesticides, accurately controlling all kinds of pests and diseases, and cultivating specialized prevention and control service organizations.

China’s Pesticide Management Regulations clearly state that highly toxic and highly toxic pesticides are prohibited from being used in vegetables, melons and fruits, tea, Chinese herbal medicines, and the relevant provisions of the Agricultural Product Quality and Safety Law and the Food Safety Law also emphasize the criminal and administrative responsibilities of prohibited pesticides. Although the government of China has taken a series of measures to reduce pesticide overuse, farmers still tend to overuse pesticides in order to increase crop yield, resulting in that the agricultural application mode has not been fundamentally changed (10–12). China is one of the largest pesticide producers and users in the world (13–15). In 2018, China consumed 1.77 million tonnes of pesticides and 46.98 million tonnes of fertilizers, which represented 42.92% and 24.92% of the world’s total consumption, respectively (FAOSTAT). Between 1990 and 2020, China’s agricultural total pesticide usage surged significantly, growing from 144.5 thousand tons to 262.7 thousand tons (16). Excessive use of pesticides not only increases the cost of agricultural production, but also causes serious pollution to the environment. In this case, how to encourage and guide farmers to reduce pesticide application has become an urgent problem to promote the sustainable development of agriculture and ensure the quality and safety of agricultural products.

With the in-depth implementation of digital village strategy, the Internet has gradually penetrated into all aspects of agriculture and rural areas, which has had a profound impact on farmers’ production and lifestyle. E-commerce is a modern business model with Internet and information technology as the core and realizing the digitalization of the whole process of goods and services transaction. Its application has widely covered retail, supply chain, cross-border trade and online and offline integration, which has significantly improved the efficiency and experience of economic activities. Under this development background, rural e-commerce, as an important extension, promotes the transformation of agriculture and rural areas with the help of e-commerce mode and infrastructure. By promoting the two-way circulation of agricultural products and industrial products, it helps upgrade agricultural industry, increase farmers’ income and optimize rural economic structure. According to the 55th Statistical Report on China’s Internet Development released by China Internet Information Center (CNNIC), by the end of 2024, the number of rural netizens had reached 313 million, the Internet penetration rate in rural areas was 65.6%, and the rural broadband access users reached 192 million. The 5G network has covered more than 96% of townships and 80% of administrative villages. The improvement of these infrastructures has significantly improved the Internet access conditions in rural areas. According to the big data monitoring of the Ministry of Commerce, in 2023, the online retail sales of rural physical goods was 1.02 trillion yuan, up 11.3% year-on-year, covering multiple fields. With the rapid development of rural e-commerce, the sales channels and circulation modes of agricultural products have changed significantly. E-commerce platform not only broadens the market scope of agricultural products, but also urges farmers to pay more attention to the quality and safety of agricultural products through information transparency and consumer feedback mechanism.

Today, the low-pesticide economic model plays a key role in market connection and production guidance in the development of China’s e-commerce and Taobao villages. On the one hand, the products produced by the low-pesticide economic model effectively connect the healthy consumer market through the accurate demand matching of e-commerce platforms, solving the problem of information asymmetry in traditional circulation. On the other hand, the market price of organic products produced by the low-pesticide economy model is higher, and farmers who are incentivized by price will be more willing to adopt the low-pesticide economy model for agricultural production.

Under the e-commerce model, the development of low-pesticide agriculture is facing multiple opportunities. Consumption upgrading and health awareness continue to expand the market demand for high-quality agricultural products, making low-pesticide products show strong growth potential (17). At the policy level, measures such as “Internet+” agricultural products out of villages and into cities and green agricultural subsidies provide systematic support for the low-pesticide model. At the same time, the Taobao village model achieves economies of scale in all links by integrating supply chain resources, significantly reducing the threshold and circulation cost of low-pesticide agricultural products entering the market.

However, the model also faces a series of real-world challenges. The primary problem is that consumer trust is difficult to establish, and the “low pesticide” standard lacks unified certification and intuitive identification methods, which can easily lead to doubts and counterfeiting. Secondly, insufficient cold chain coverage and high transportation costs still restrict business viability in many rural areas. Furthermore, smallholder farmers generally face insufficient e-commerce capabilities, including a shortage of digital skills such as online store maintenance, content marketing, and customer service (18). Therefore, how to achieve sustainable commercialization and large-scale low-pesticide economic models still need to be explored in depth.

Above phenomenon provides a unique research background for studying the influence of ECA on FPRB. In order to clarify its effect, this paper needs to solve the following questions: Can ECA promote FPRB? If so, what is the influencing mechanism? Is there heterogeneity in the influence of ECA on FPRB? Therefore, this paper takes 3531 farmers in 10 provinces of China as the research objects, and systematically studies the influence of ECA on FPRB.

## 2 Literature review

Farmers are the key subjects of pesticide application, and pesticide application is an important link in agricultural production. Previous studies have explored the deep-seated reasons of farmers’ pesticide application behavior through their pesticide application appearances, and conducted a lot of research on the influencing factors of farmers’ pesticide application behavior, which are mainly divided into internal factors and external factors.

From the internal factors, the education level of farmers (19–21), family income (20, 22), awareness of pesticide residues (22), awareness of environmental pollution (22) and awareness of safe production (22) will have an impact on farmers’ pesticide application behavior. For example, Sharma et al. (19) and Pan et al. (21) pointed out in their research that the higher the education level of farmers, the more likely they are to use pesticides according to the instructions, thus reducing pesticide application. On the other hand, Ali et al. (20) found that vegetable farmers with higher knowledge and income would prefer to use more pesticides to ensure high yield and high income of crops based on the data of 917 households in Bangladesh. In addition, Li et al. (22) found that the influence of farmers’ awareness of environmental pollution and pesticide residues on their environmentally friendly pesticide application behavior increased with time.

From the external factors, government subsidies (22), government publicity and education (22) and technical training (21, 23), the role of suppliers (21), social norms (24), weather conditions (25, 26), agricultural machinery socialization service (27) and other factors will have an impact on farmers’ pesticide application behavior. Li et al. (22) found that government subsidies and government publicity and education can effectively promote farmers to implement environmentally friendly pesticide application behavior. Jallow et al. (23) found that providing external support and technical training for farmers in pesticide application can help farmers to master more knowledge and adopt new technologies or alternative pest control methods with a more positive attitude, thus reducing excessive use of pesticides. Wang et al. (24) found that farmers will consider reducing pesticide application if they feel the pressure from the surrounding people such as family, friends and neighbors and are subject to invisible social norms. Koleva and Schneider (25), when analyzing the situation in the United States and Malaysia, found that the increase of temperature and rainfall will make the pest attack more serious, which will lead to the increase of pesticide use by farmers. In addition to the application amount, weather conditions will also affect the time and frequency of pesticide application by farmers (26). Zhang et al. (27) found that the use of agricultural machinery helps to improve pesticide spraying efficiency, thus prompting small farmers to reduce pesticide application.

In recent years, scholars have paid attention to the influence of e-commerce on agricultural sustainable production. For example, Wang et al. (28) based on the data of 733 fruit farmers in rural areas of China, discussed the influence of e-commerce participation on the use intensity of organic fertilizer for fruit farmers, and found that the participation of e-commerce greatly increased the amount of organic fertilizer input. Compared with fruit farmers with low participation in e-commerce, fruit farmers with high participation in e-commerce use more organic fertilizers. Zhao et al. (29) and others discussed the influence of e-commerce participation on the adoption of sustainable agricultural technology by immigrants in the Three Gorges reservoir area of China, and confirmed that e-commerce participation promoted the adoption of sustainable agricultural technology, and the expectation of ecological value of agricultural products and agricultural technical support played an important driving role in it. Wang et al. (30) took the national e-commerce demonstration city as a quasi-natural experiment, and studied the impact of urban e-commerce development on agricultural non-point source pollution and its mechanism by using the multi-stage difference method (DID). It was found that the total consumption of chemical fertilizers in the pilot cities of the national e-commerce demonstration policy decreased by 7.5 percentage points, and it also inhibited the consumption of nitrogen fertilizer, pesticides and agricultural film. Qiu et al. (31) used the sample of 2112 farmers in Jiangsu, China Province to explore the relationship between ECA and farmers’ adoption of sustainable production technology, and found that ECA promoted farmers’ adoption of sustainable production technology by enhancing their technological cognition.

To sum up, some studies have paid attention to the role of e-commerce in promoting the sustainable transformation of agricultural production, which laid a good foundation for this paper, but there is still room for further research. Most studies focus on the sustainable technology adoption effect and chemical fertilizer reduction effect of e-commerce, but lack of systematic investigation on its pesticide reduction effect, which limits the understanding of the sustainable development effect of e-commerce. Therefore, based on the national micro-survey data of farmers, this paper explores the pesticide reduction effect of e-commerce, and further discusses the influence differences of different ECA scales, operation models and pesticide application models, which is helpful to formulate more targeted pesticide reduction policies from different perspectives.

## 3 Theoretical framework

### 3.1 The direct impact of ECA on FPRB

E-commerce adoption has significantly promoted FPRB through information transmission, market incentives and technical empowerment.

First of all, the e-commerce platform, as an information dissemination medium, provides important support for farmers to reduce pesticides. Information symmetry is an important basis for pesticide reduction (32). Farmers often face the problem of insufficient information in the decision-making of pesticide use, and the information transmission function of e-commerce platform can effectively alleviate this problem, so that farmers can more clearly understand the benefits of pesticide reduction and enhance their willingness to reduce pesticides. On the one hand, the e-commerce platform helps farmers to better understand sustainable prevention and control technologies, improve farmers’ awareness of sustainable production, and enhance farmers’ willingness to adopt integrated pest control and sustainable prevention and control technologies, thus promoting them to reduce pesticide use in the production process. On the other hand, by integrating diversified information resources, the e-commerce platform enables farmers to systematically understand the potential impact of pesticide use on the ecological environment, the quality and safety of agricultural products and human health, thus promoting the transformation of their pesticide use behavior to a more scientific and standardized direction (33).

Secondly, the e-commerce platform reconstructs the circulation system of agricultural products, which provides a positive market incentive for farmers to reduce pesticides and urges them to reduce pesticides. E-commerce platform establishes a direct channel of “production-consumption” by shortening the intermediate circulation channels, so that farmers can directly face end consumers and understand consumers’ needs and preferences for sustainable agricultural products. Consumers’ preference and price premium for agricultural products with low pesticide residues provide economic incentives for FPRB to meet market demand and obtain higher income (34). For farmers, the sales price of sustainable agricultural products on e-commerce platform is generally higher than that of traditional channels. This significant price difference strengthens the motivation of farmers to adopt sustainable production methods, and then urges them to actively reduce the use of pesticides to obtain higher market income.

Finally, the e-commerce platform provides all-round technical support for FPRB by integrating professional resources. E-commerce platform can integrate resources such as agricultural technical experts, sustainable prevention and control technology suppliers and professional training institutions, and provide farmers with one-stop services from pest monitoring, sustainable prevention and control technology guidance to pesticide safety use training, thus alleviating the technical obstacles of pesticide reduction for farmers and reducing the production risks caused by improper use of pesticides. At the same time, the technical support provided by the e-commerce platform also has a demonstration effect, which can promote the spread and application of sustainable prevention and control technologies, promote environmentally-friendly pesticides with high efficiency, low toxicity and low residue, and significantly improve the scientific and effective use of pesticides. In addition, the e-commerce platform can use big data analysis technology to provide customized pest control programs for farmers, further optimize the efficiency of pesticide use, and achieve the goal of pesticide reduction (35, 36).

Based on the above analysis, this paper puts forward research hypothesis 1:

H1: ECA has a significant positive impact on FPRB.

### 3.2 The impact mechanism of ECA on FPRB

#### 3.2.1 The mediating role of agricultural income expectations

Based on bounded rationality theory, farmers will pursue the maximization of interests in the decision-making process, and make rational analysis according to the existing information, so as to determine the optimal scheme within the cognitive range. Pesticide reduction not only affects the quantity and quality of agricultural products, but also directly relates to the production cost and household income of farmers. Therefore, in the decision-making process, farmers will rationally evaluate the effectiveness of pesticide reduction, with the goal of maximizing income and minimizing risk, and form expectations for agricultural income. When farmers expect pesticide reduction to bring higher agricultural income, their willingness to adopt pesticide reduction technology will be significantly enhanced. On the contrary, if the expected income is low or the risk is high, farmers may tend to maintain the status quo.

E-commerce adoption has significantly improved farmers’ agricultural income expectations, and thus achieved pesticide reduction. E-commerce platform provides farmers with key data such as market supply and demand information, price fluctuation trend and consumer preferences, which helps farmers to predict agricultural income more accurately, and directly improves the market competitiveness and premium ability of agricultural products by reducing intermediate links and broadening sales channels, thus enhancing farmers’ optimistic expectations of agricultural income (37), enabling farmers to more clearly foresee the economic benefits brought by pesticide reduction (38). At the same time, the technical training and support provided by the e-commerce platform further reduces the risk of FPRB, making them more inclined to achieve income growth through technological innovation.

Based on the above analysis, this paper puts forward research hypothesis 2:

H2: ECA promotes FPRB by raising agricultural income expectation.

#### 3.2.2 The mediating role of food safety perception

Awareness and cognition are important factors affecting farmers’ adoption decision (39–41). If farmers do not understand the role of the agricultural technology sufficiently, they may be less likely to adopt it (42). Therefore, food safety perception has a significant impact on FPRB. Food safety perception reflects farmers’ understanding and attention to the safety production standards of agricultural products, which is an important internal driving force for farmers’ technical behavior choice (43). With the increasing demand of consumers for food safety, farmers’ perception of safety production technology directly affects their production decisions. When farmers have a higher perception of food safety, they will be more inclined to adopt technical behaviors that can ensure the safety of agricultural products, such as pesticide reduction technology, to meet market demand and enhance their competitiveness. In addition, food safety perception can also standardize farmers’ production behavior and urge them to actively reduce pesticide use in the production process to meet the safety production standards (44).

E-commerce adoption has significantly improved farmers’ perception of food safety, and thus achieved pesticide reduction. E-commerce platform provides farmers with information on consumers’ demand for food safety, market access standards and related policies and regulations, helping farmers to understand the importance of food safety more comprehensively. At the same time, through the establishment of a transparent supply chain system, the e-commerce platform enables farmers to directly perceive the role of safe production technology in improving the market competitiveness of products, and realize the positive role of pesticide reduction in ensuring food safety and enhancing product value, thus enhancing their willingness to reduce.

Based on the above analysis, this paper puts forward research hypothesis 3:

H3: ECA promotes FPRB by improving food safety perception.

#### 3.2.3 The mediating role of adoption of agricultural machinery socialization services

The socialized service of agricultural machinery plays an important role in farmers’ decision on pesticide reduction and is an important way to promote the sustainable transformation and sustainable development of agriculture (45). First of all, the socialization service of agricultural machinery can accurately control the application amount of pesticides and effectively reduce the abuse of pesticides by introducing specialized agricultural machinery and technologies, such as drone plant protection and precision spraying system (46, 47). Secondly, the socialization service of agricultural machinery can improve agricultural production efficiency by reducing farmers’ production cost and time cost, and then promote farmers to adopt pesticide reduction technology (48). This service model reduces the burden of farmers on equipment purchase, maintenance and labor input, and improves the utilization efficiency of pesticides through specialized operation. Finally, the socialization service of agricultural machinery ensures the scientificity and effectiveness of pesticide reduction measures through standardized service process and professional management, improves the attach rate and utilization rate of pesticides, and reduces the waste and abuse of pesticides.

E-commerce adoption has significantly promoted the adoption of socialized agricultural machinery services by farmers, and thus achieved pesticide reduction. E-commerce platform provides farmers with information about agricultural machinery socialization service, including service content, technical advantages and cost-benefit analysis (46), which promotes farmers’ willingness to adopt agricultural machinery socialization service. At the same time, the e-commerce platform reduces the information asymmetry by establishing the direct connection between farmers and agricultural machinery service providers, and enables farmers to obtain specialized services more conveniently (45). With the support of e-commerce platform, farmers can obtain specialized agricultural machinery services more efficiently, and reduce pesticide consumption through precise operation, thus achieving pesticide reduction.

Based on the above analysis, this paper puts forward research hypothesis 4:

H4: ECA promotes FPRB by promoting the adoption of agricultural machinery socialization services.

**FIGURE 1:**
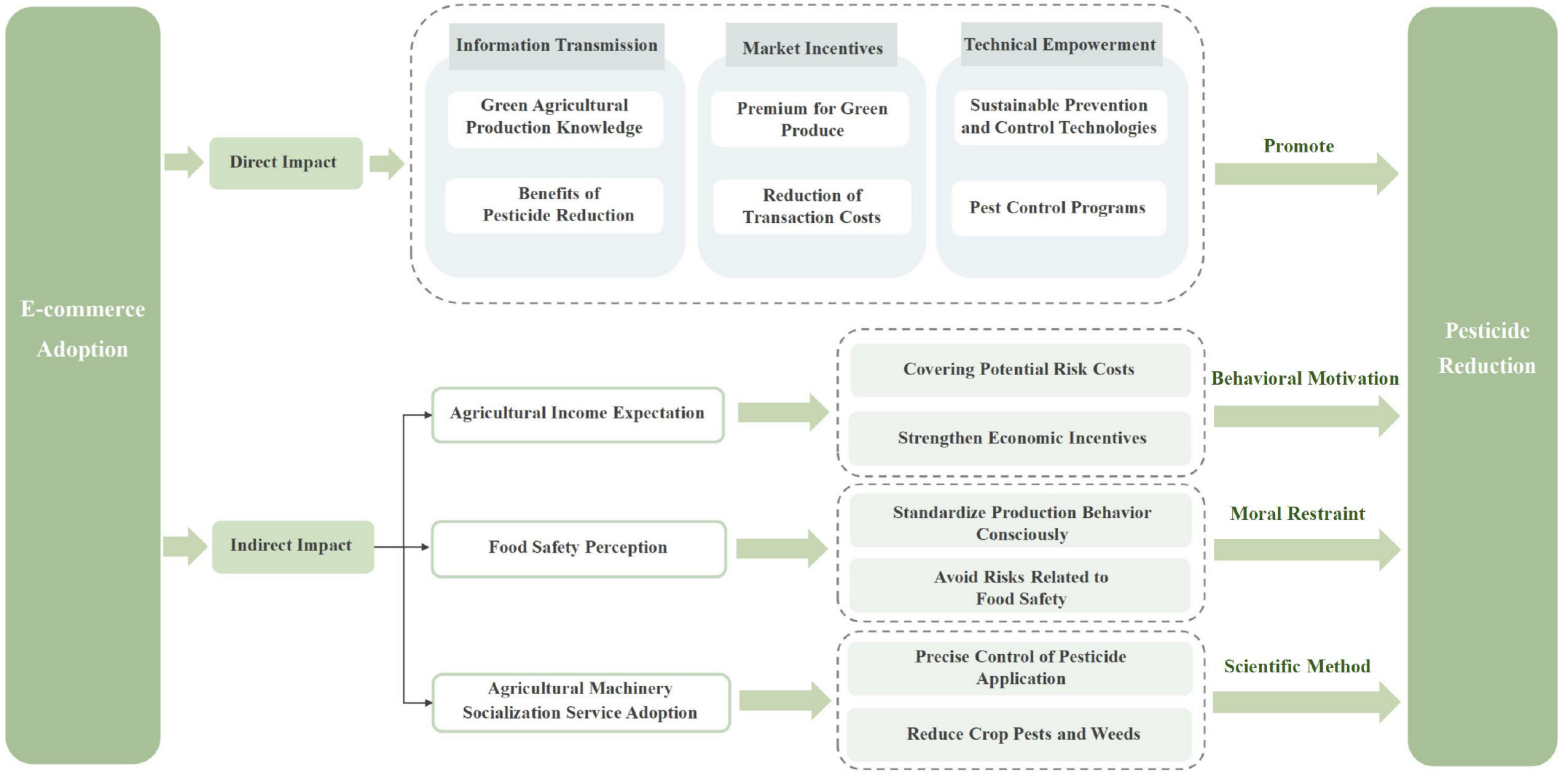
Theoretical framework.

The theoretical analysis framework of this paper is shown in Figure 1.

## 4 Materials and methods

### 4.1 Data sources

The data used in this paper comes from the China Rural Revitalization Survey (CRRS), the nationwide large-scale rural follow-up survey conducted by the Rural Development Institute of Chinese Academy of Social Sciences in 2020. The database comprehensively considers the development level and geographical distribution of county economy, conducts field research according to the per capita GDP of each province and county, the economic development of each township and village in the investigated county, and the principle of full coverage of space, and adopts the method of combining stratified sampling, equidistant sampling and random sampling. CRRS database covers ten provinces in the east, middle, west and northeast, Shandong, Zhejiang and Guangdong in the east, Anhui and Henan in the middle, Sichuan, Guizhou, Shanxi and Ningxia Hui Autonomous Region in the west and Heilongjiang in the northeast. The fields involved include farmers’ income and expenditure, agricultural production structure, production mode, production environment, farmers’ financial market participation, informationization and e-commerce development, which have been widely used and have strong representation. After eliminating the missing and abnormal values of key variables, a total of 3531 samples were retained for research.

**FIGURE 2:**
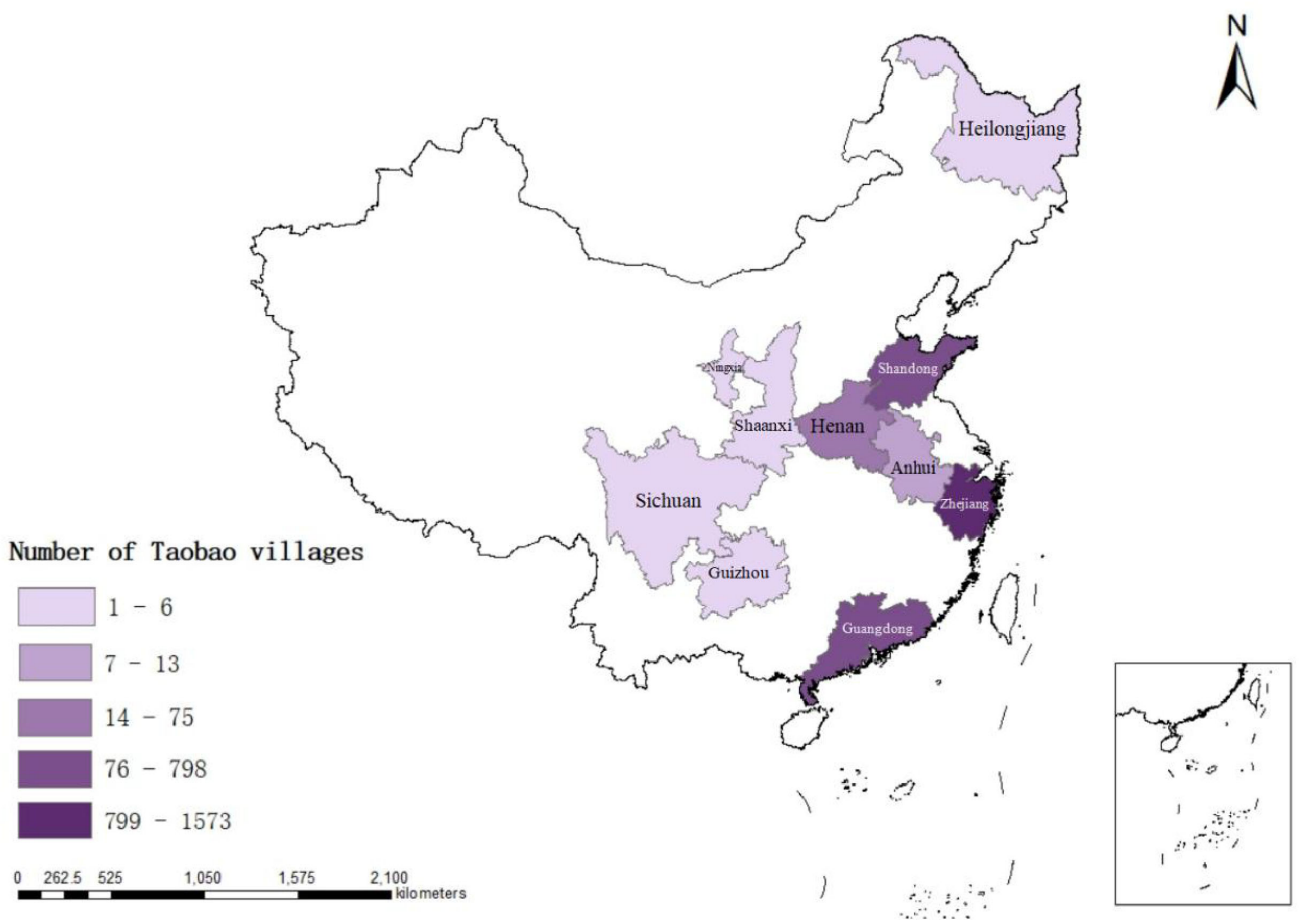
Distribution of Taobao villages in sample provinces.

According to the data of Taobao Village in China from 2009 to 2022 published by Ali Research Institute, this paper selects the data of Taobao Village in the same period as the survey sample, and draws the development of e-commerce in the surveyed provinces, as shown in Figure 2. As can be seen from Figure 2, the number of Taobao villages in Shandong Province, Zhejiang Province and Guangdong Province in the eastern region is obviously higher than that in the central, western and northeastern provinces. The possible reason is that the economy in the eastern region is more developed, the infrastructure of ECA is more perfect, and the level of e-commerce development is higher.

### 4.2 Variable selection

#### 4.2.1 Dependent variable

The dependent variable of this paper is FPRB. The questionnaire inquired in detail about the changes in the average pesticide use per hectare of farmers compared with 5 years ago. Therefore, select the questionnaire “Compared with 5 years ago, does the average pesticide use per hectare decrease?” This problem is used to measure FPRB, and the sample that answers “reduction” is assigned to 1, otherwise it is assigned to 0.

#### 4.2.2 Core independent variable

The core independent variable of this paper is ECA. Using the research of Qiu et al. (49) for reference, this paper selects the question “Does your family operate products through online transactions” in the questionnaire to measure the ECA of farmers. If farmers sell products through e-commerce, the value is 1, otherwise the value is 0.

#### 4.2.3 Intermediate variables

Based on the theoretical analysis above, it can be seen that ECA can promote FPRB by enhancing agricultural income expectations, enhancing food safety perception and adopting agricultural machinery socialization services. This paper selects the questionnaire “How do you think your family’s agricultural income level will change in 2020 compared with that in 2019?” This indicator is used to measure farmers’ agricultural income expectation. If the answer is “increased” or “increased more,” it is assigned to 1, otherwise it is assigned to 0. At the same time, this paper uses the questionnaire “Do you usually care about food safety?” This question is used to measure farmers’ perception of food safety. The answer to “very concerned” or “relatively concerned” is assigned to 1, otherwise it is assigned to 0. In addition, this paper measures the adoption of agricultural machinery socialization service by the question of “mode of pesticide application” in the questionnaire. If you buy machinery service to apply pesticide, it is assigned to 1, otherwise it is assigned to 0.

#### 4.2.4 Instrumental variable

Referring to the research of Su (50), this paper uses the spherical distance from Hangzhou to cities as a tool variable. The e-commerce represented by Taobao originated in Hangzhou, so Hangzhou is in a leading position in the development of e-commerce. Generally speaking, the closer to Hangzhou geographically, the stronger the e-commerce radiation-driven effect on the city, and the higher the degree of e-commerce development. From the perspective of correlation, Hangzhou, as the core area of e-commerce development, has a significant radiation effect on the ECA in the surrounding areas. There is a strong correlation between the spherical distance from Hangzhou to cities and the development degree of ECA, which can effectively reflect the geographical differences of ECA. From the externality point of view, the spherical distance is a geographical objective index, which is not directly affected by economic activities or policy factors, and has nothing to do with the error term in the model, and meets the exogenous requirements of tool variables. At the same time, the spherical distance only indirectly affects the pesticide reduction by affecting the radiation effect of ECA, but does not directly affect the pesticide reduction, which is in line with exclusive constraints. Therefore, the spherical distance from Hangzhou to cities is a reasonable tool variable, which can effectively solve the possible endogenous problems between ECA and pesticide reduction.

#### 4.2.5 Control variables

In addition to ECA, individual characteristics, family characteristics and village characteristics of farmers will all affect FPRB to some extent. Referring to the existing research (20, 21), this paper selects control variables from three dimensions: individual characteristics, family characteristics and village characteristics. Among them, individual characteristics include gender, age, education level and marital status; Family characteristics include family agricultural insurance, family political identity and family cultivated land scale; the characteristics of a village include its topography, its distance from the county government and its economic development level.

Table 1 shows the definition, assignment and statistical characteristics of the above variables.

**TABLE 1:** Definition of variables and descriptive statistical results.

Variable type	Variable name	Variable definition and assignment	Mean value	Standard deviation
Dependent variable	FPRB	Compared with 5 years ago, has the use of pesticides decreased: 1 = Yes, 0 = No	0.711	0.453
Core independent variable	ECA	Does your family operate products that are traded through the Internet: 1 = Yes, 0 = No	0.100	0.300
Intermediate variables	Agricultural income expectations	Do you think the agricultural income level of your family in 2020 is higher than that in 2019: 1 = Yes, 0 = No	0.743	0.437
	Food safety perception	Do you usually care about food safety? 1 = Care, 0 = Don’t care	0.931	0.254
	Agricultural machinery socialization service adoption	Whether the socialized service of agricultural machinery is adopted in the pesticide spraying link: 1 = Adopted, 0 = Not adopted	0.134	0.341
Instrumental variable	The spherical distance from Hangzhou to each city	Calculated according to the actual value, unit: Km, taking logarithm	6.706	1.037
Control variables	Gender	1 = Male, 0 = Female	0.932	0.251
	Age	Calculated by actual age, unit: years	59.799	11.264
	Degree of education	1 = Primary school and below, 2 = Junior high school, 3 = High school (technical secondary school, vocational high school), 4 = University or above	1.757	0.727
	Marital status	1 = Married, 0 = Other	0.914	0.281
	Family agricultural insurance	Does your family buy agricultural insurance: 1 = Yes, 0 = No	0.806	0.395
	Family political identity	Whether there is party member in the family: 1 = Yes, 0 = No	0.225	0.418
	Family cultivated land scale	Calculated according to actual value, unit: hectare, taking logarithm	1.228	3.703
	Village topography	1 = Plain, 2 = Hill, 3 = Mountain	1.924	0.873
	Distance from the village to the county government	Calculated according to actual value, unit: Km	23.279	17.278
	Village economic development level	Per capita net income of your village, unit: CNY, taking logarithm	9.427	0.538

**FIGURE 3:**
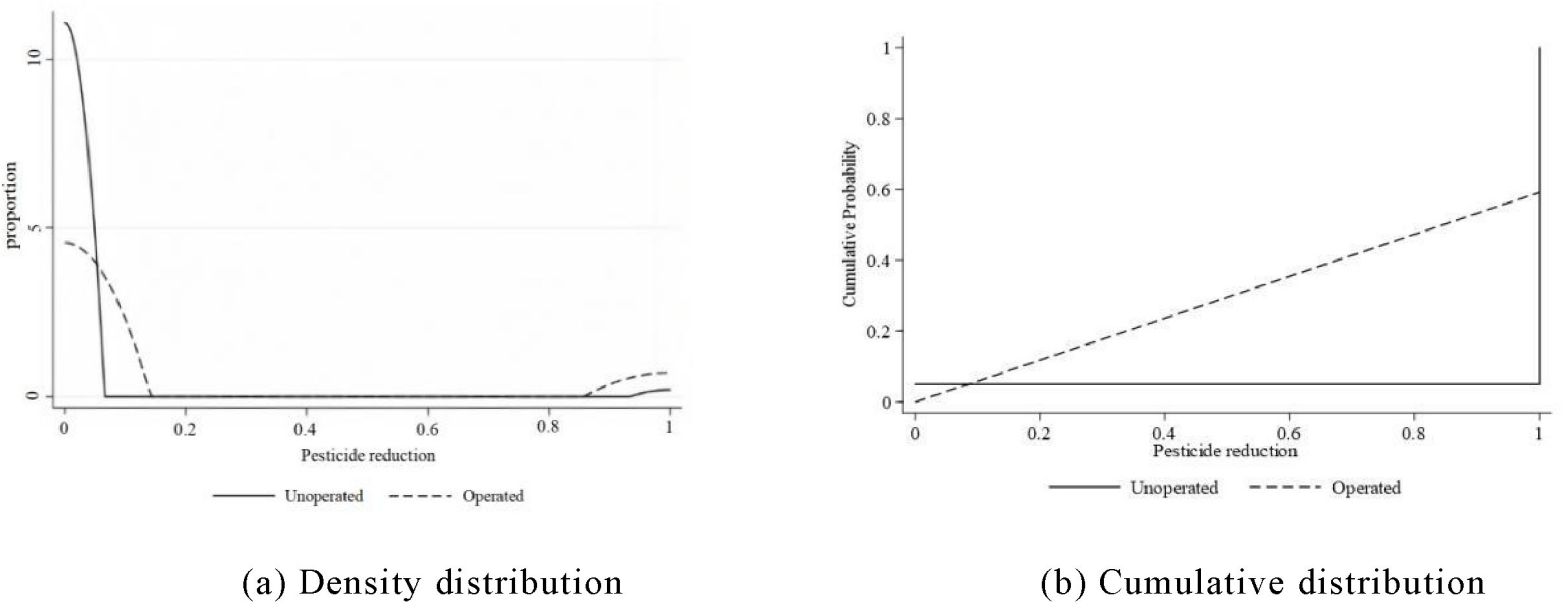
**(a,b)** Density distribution and cumulative distribution of FPRB.

This paper compares the pesticide reduction of e-commerce farmers and non-e-commerce farmers. Figures 3a, b shows the density distribution and cumulative distribution of pesticide reduction, respectively. It can be seen from the figure that the probability of pesticide reduction by e-commerce farmers is higher than that of non-e-commerce farmers. From this, it can be preliminarily judged that ECA plays an important role in promoting pesticide reduction. Of course, to confirm the impact of e-commerce on pesticide reduction, more rigorous quantitative analysis is needed. The following will empirically analyze the impact of ECA on FPRB.

### 4.3 Model construction

#### 4.3.1 Benchmark regression model

Considering that the dependent variable “FPRB” is a binary classification variable, this paper uses binary Logit model for regression analysis, and the model is set as follows:


(1) $$F\,P\,R\,B=\beta_{0}+\beta_{1}\,E\,C\,A+\beta_{2}\,X+\varepsilon$$

In the formula (Equation 1), *FPRB* represents the farmers’ pesticide reduction behavior; *ECA* represents e-commerce adoption; *X* is the control variables, including the individual characteristics, family characteristics and village characteristics of farmers; β_1_ and β_2_ are the coefficient to be estimated; β_0_ is constant term; ε is error term.

#### 4.3.2 Intermediary effect model

Referring to the research (49) of this paper uses the intermediary effect model to test the mechanism of agricultural income expectations, food safety perception and agricultural machinery socialization service adoption. The model formula (Equations 2–4) are as follows:


(2) $$F\,P\,R\,B=\lambda_{1}+c\,E\,C\,A+\alpha_{1}\,X+\mu_{1}$$

(3) $$M=\lambda_{2}+a\,E\,C\,A+\alpha_{2}\,X+\mu_{2}$$

(4) $$F\,P\,R\,B=\lambda_{3}+c^{\prime }\,E\,C\,A+b\,M+\alpha_{3}\,X+\mu_{3}$$

In the formula, *FPRB* represents the farmers’ pesticide reduction behavior; *ECA* represents e-commerce adoption; *M* represents agricultural income expectations, food safety perception and agricultural machinery socialization service adoption; *X* is the control variables, including the individual characteristics, family characteristics and village characteristics of farmers; *c*, *a*, *c*′, α1, α2, α3, *b* represent the coefficient to be estimated; λ1, λ2, λ2 represent constant term; μ1, μ2, μ3 represent error term.

## 5 Results and analysis

### 5.1 Benchmark regression results

Table 2 shows the results of benchmark regression. Column (1), column (2) and column (3) are the results of gradually adding individual characteristics, family characteristics and village characteristics of farmers, and column (4) is the result of marginal effect. From the results of column (3) and column (4), it can be seen that the influence of ECA on FPRB is positively significant at the statistical level of 1%, indicating that ECA has a significant positive influence on FPRB, and ECA will increase the probability of farmers’ pesticide reduction by 38.3%, and the research hypothesis 1 is verified. The reason is that ECA has lowered the threshold for farmers to acquire sustainable production knowledge, improved the market income of sustainable products, eased the technical obstacles of pesticide reduction, and promoted farmers to reduce pesticide application.

**TABLE 2:** Benchmark regression results.

Variable name	(1)	(2)	(3)	(4)
ECA	1.151*** (0.115)	1.191*** (0.117)	1.236*** (0.117)	0.383*** (0.035)
Gender	0.058 (0.095)	0.063 (0.096)	0.048 (0.097)	0.015 (0.030)
Age	−0.007*** (0.002)	−0.007*** (0.002)	−0.006** (0.002)	−0.002** (0.001)
Degree of education	0.154*** (0.030)	0.133*** (0.031)	0.164*** (0.032)	0.051*** (0.010)
Marital status	0.159* (0.087)	−0.151* (0.088)	−0.103 (0.089)	−0.032 (0.028)
Family agricultural insurance		0.485*** (0.057)	0.449*** (0.057)	0.139*** (0.017)
Family political identity		0.224*** (0.054)	0.232*** (0.055)	0.072*** (0.017)
Family cultivated land scale		0.006 (0.007)	0.005 (0.007)	0.002 (0.002)
Village topography			−0.332*** (0.049)	−0.103*** (0.015)
Distance from the village to the county government			0.001 (0.001)	0.001 (0.001)
Village economic development level			−0.288*** (0.044)	−0.089*** (0.013)
Constant term	0.707*** (0.181)	0.299 (0.192)	2.983*** (0.465)	3531
Pseudo R^2^	0.044	0.062	0.087	
N	3531	3531	3531	

***,

** and

*Mean significant at the statistical level of 1%, 5% and 10% respectively, and the figures in brackets are standard errors, the same below.

In terms of individual characteristics of farmers, age has a significant negative impact on FPRB. The possible reason is that elderly farmers have long maintained traditional agricultural production methods and are used to high-intensity application of pesticides to ensure agricultural production. Moreover, most elderly farmers have low education level and weak ecological concept, and are less aware of the environmental harm caused by excessive application of pesticides. Education level has a significant positive impact on FPRB. The possible reason is that farmers with high education level are more likely to understand the harm of excessive use of pesticides to environmental factors such as soil and water and the quality and safety of agricultural products, and are more likely to actively reduce pesticide use. In addition, from the point of view of information acquisition, farmers with high education are better at obtaining accurate agricultural indexes through various channels, including efficient use of pesticides and ecological prevention measures of pests, which enables them to reduce pesticide consumption while ensuring yield.

In terms of family characteristics of farmers, family agricultural insurance has a significant positive impact on FPRB. The possible reason is that after participating in agricultural insurance, the natural risks and market risks faced by farmers are effectively shared, reducing the dependence on excessive application of pesticides to ensure production and reduce risk losses. Family political identity has a significant positive impact on FPRB. The possible reason is that party member at home can obtain advanced agricultural technology knowledge and relevant national pesticide application norms more conveniently through the learning activities of party organizations in time, and actively publicize advanced technology and application norms to family members to guide them to practice pesticide reduction activities in agricultural production.

In terms of farmers’ village characteristics, village topography has a significant negative impact on FPRB. The possible reason is that mountain agriculture relies more on traditional farming methods and tends to overuse pesticides to ensure agricultural output, and the low penetration rate of e-commerce in complex terrain areas also indirectly affects FPRB. The level of village economic development has a significant negative impact on FPRB. The possible reason is that villages with higher economic development level often turn to planting high-value cash crops such as high-end fruits and flowers, which are more sensitive to pests and diseases and have strict market requirements for quality. In order to ensure high income, farmers tend to increase pesticide input to ensure yield and appearance quality.

### 5.2 Robustness test

#### 5.2.1 Replacement estimation method

In order to ensure the robustness of the benchmark regression results, this paper replaces the binary Logit model with the binary Probit model, and then carries out the estimation test again. The results are shown in column (1) of Table 3. After replacing the model, ECA still has a significant positive impact on FPRB, and the previous estimation results are robust.

**TABLE 3:** Robustness test results.

Variable name	(1)	(2)	(3)	(4)
	Replacement estimation method	Replacement sample values	Replacement dependent variables	Tail shrinking treatment
ECA	2.329*** (0.250)	1.196*** (0.172)	−0.551*** (0.137)	1.244*** (0.117)
Control variables	Yes	Yes	Yes	Yes
Constant term	4.884*** (0.781)	2.450*** (0.670)	3.479***	3.339*** (0.490)
N	3531	1893	3531	3531

***Means significant at the statistical level of 1%.

#### 5.2.2 Replacement sample values

Considering the particularity of farmers over 60 years old in physical fitness and health status, this paper deletes the samples of farmers over 60 years old and regresses them to eliminate the interference of some special factors and make the results more representative. The results are shown in column (2) of Table 3. ECA still has a significant positive impact on FPRB, which further verifies the robustness of the previous results.

#### 5.2.3 Replacement dependent variables

In addition, this paper regresses by replacing the dependent variable to verify the robustness of the previous results. In the benchmark regression, “whether the use of pesticides is reduced compared with 5 years ago” is used to measure FPRB, which is replaced by “the cost of self-purchased preventive pesticides by farmers” and re-estimated. The result is shown in column (3) of Table 3. ECA has a significant negative impact on farmers’ self-purchasing cost of disease prevention pesticides, which indicates that ECA promotes FPRB, is conducive to reducing pesticide application and promoting agricultural sustainable production, further confirming the robustness of the previous results.

#### 5.2.4 Tail shrinking treatment

In order to avoid the influence of extreme values on the regression results, 1% of the data at the left and right ends of continuous variables in the controlled variables are truncated and then regressed. The results are shown in column (4) of Table 3. ECA still has a significant positive impact on FPRB, and the above estimation results are robust.

#### 5.2.5 Placebo test

**FIGURE 4:**
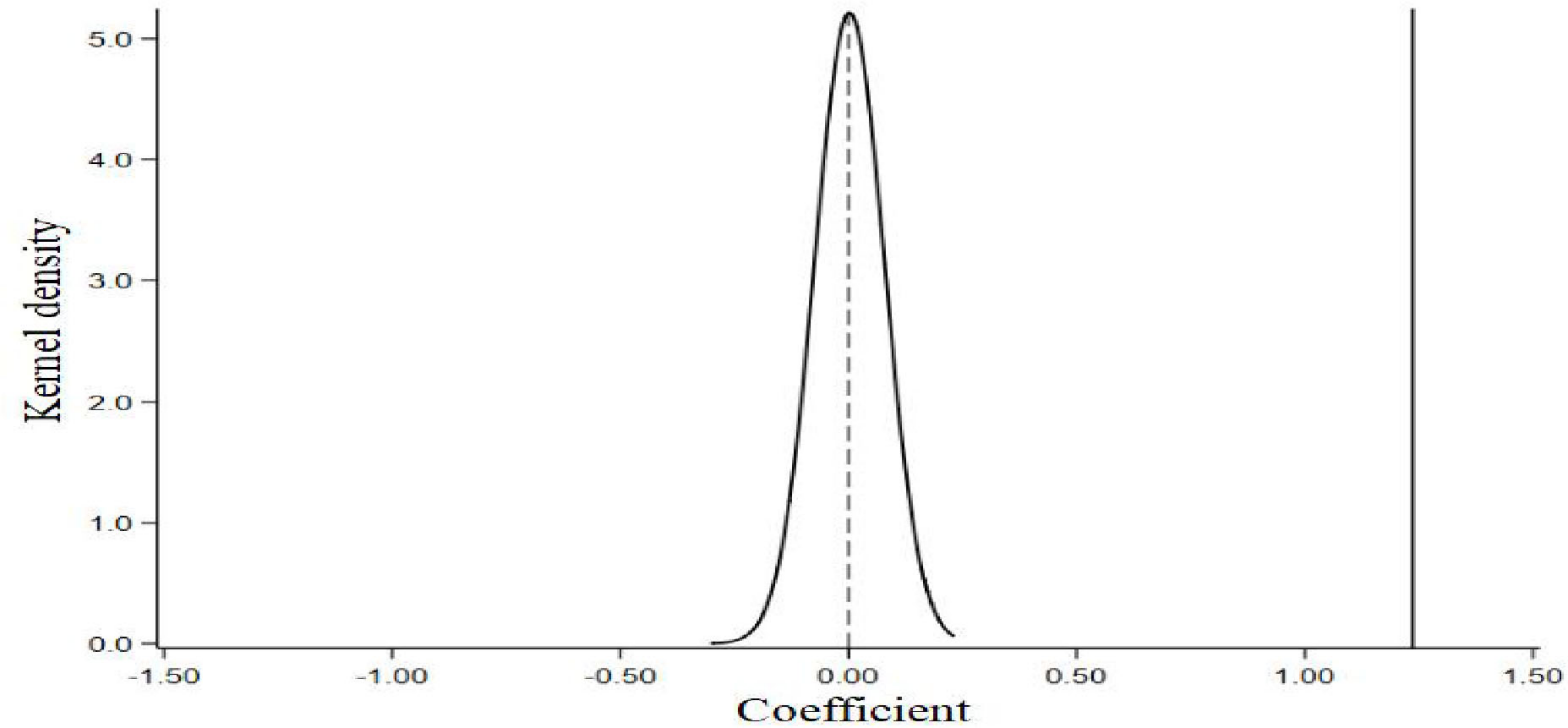
Placebo test.

In order to verify whether the unobserved random factors will affect the regression results, this paper set up an experimental group to carry out placebo test, and the remaining samples were used as the control group. As shown in Figure 4, after repeating the randomization operation for 1000 times, the average value of the regression coefficient of ECA approaches to zero, and presents the characteristics of approximate normal distribution. In addition, this average value is significantly different from the estimated value in the benchmark regression model. Therefore, the potential interference caused by unobserved factors can be ruled out, which indirectly proves that ECA can significantly promote FPRB.

### 5.3 Endogenous test

#### 5.3.1 Self-selection deviation

Considering that the behavior decision of ECA is not random, it may be influenced by other factors such as individual, family or village characteristics. Therefore, in order to avoid the estimation bias caused by self-selection, this paper uses the propensity score matching method (PSM) to re-estimate. The average treatment effect of ECA on FPRB is shown in Table 4. ECA has a significant positive impact on FPRB, whether it is estimated by the propensity score matching methods such as nearest neighbor matching, radius matching, kernel matching, mahalanobis matching or local linear matching.

**TABLE 4:** Propensity score matching method (PSM) estimation results.

Variable name	Matching method	Experimental group	Control group	ATT	Std.	*T*-value
ECA	Nearest neighbor matching	0.949	0.656	0.293	0.031	9.53
	Radius matching	0.949	0.685	0.264	0.014	18.97
	Kernel matching	0.949	0.667	0.282	0.015	18.66
	Mahalanobis matching	0.949	0.645	0.324	0.034	9.97
	Local linear matching	0.949	0.669	0.280	0.030	9.24

**FIGURE 5:**
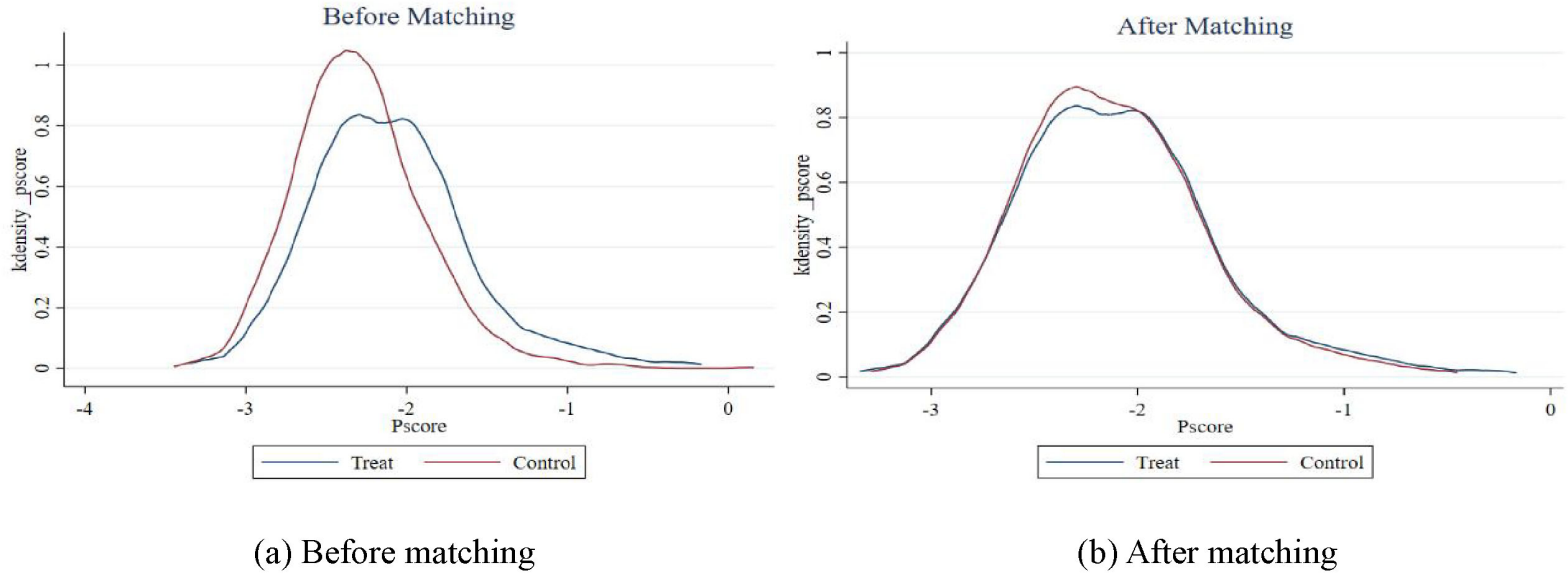
**(a,b)** Common support domain before and after matching.

The PSM model specification must meet two preconditions: overlapping assumption and balanced characteristics. For the overlapping hypothesis, it is necessary to test the common support domain. After the matching is completed, if the area of the common support domain is too small, it means that there are too many sample losses and the matching effect is not good. On the contrary, it means that the matching result is good. Figure 5 is a trend score distribution diagram of the test common support domain. The area of common support domain after matching Figure 5a is larger than that before matching Figure 5b, and there is a large common support interval, which shows that the matching effect is good and the common support domain test passes.

At the same time, in order to examine whether the above propensity score matching estimation results balance the data well, it is necessary to carry out a balance test, and the balance test results are shown in Table 5. In this paper, the kernel matching method is taken as an example. The results show that the standard deviation rate of most control variables is reduced after matching, and the standard deviation rate is less than 10%. At the same time, most of the *t*-test results of control variables do not reject the original assumption that there is no systematic difference between the experimental group and the control group, which effectively balances the difference of covariate distribution between the experimental group and the control group. Therefore, the matching results of propensity score pass the balance test.

**TABLE 5:** Balance test results.

Variable name	Mean value before matching		Mean value after matching		Deviation rate (%)		T-test after matching	
	Experimental group	Control group	Experimental group	Control group	Before	After	T	P > t
Gender	0.906	0.935	0.906	0.917	−10.6	−4.2	−0.53	0.596
Age	61.455	59.616	61.422	60.948	15.9	4.3	0.56	0.572
Degree of education	1.770	1.755	1.772	1.771	2.0	0.2	0.03	0.979
Marital status	0.898	0.916	0.897	0.902	−6.2	−1.6	−0.21	0.836
Family agricultural insurance	0.733	0.814	0.732	0.765	−19.5	−7.9	−1.00	0.317
Family political identity	0.196	0.229	0.197	0.203	−8.0	−1.6	−0.22	0.826
Family cultivated land scale	1.870	1.157	1.730	1.425	14.7	6.3	0.84	0.402
Village topography	1.849	1.932	1.849	1.875	−9.7	−3.0	−0.40	0.686
Distance from the village to the county government	22.845	23.327	22.854	22.886	−2.9	−0.2	−0.03	0.979
Village economic development level	9.581	9.410	9.578	9.540	31.0	7.1	0.86	0.391

#### 5.3.2 Reverse causality

There may be endogenous problems caused by reverse causality in benchmark regression, which makes the previous conclusions biased. On the one hand, ECA promotes FPRB through economic incentives and the spread of sustainable production concepts; On the other hand, after pesticide reduction, the economic value of sustainable and high-quality agricultural products in the market is higher, and farmers are more likely to carry out ECA activities to expand the scope of market transactions and increase income.

In order to solve this endogenous problem and improve the robustness of the model estimation, this paper replaces the measurement index of FPRB with the cost of self-purchased preventive drugs by farmers, and uses two-stage lease squares (2SLS) and Extended regression model (ERM) to estimate. Referring to the research of Su and Cao (51), this paper selects the spherical distance from Hangzhou to each city as a tool variable for ECA. From the correlation point of view, this tool variable is closely related to ECA. As the birthplace of e-commerce in China, generally speaking, the closer to Hangzhou, the greater the radiation-driven effect, and the greater the probability of ECA for farmers in this area; from the exogenous point of view, it is difficult for this tool variable to directly affect FPRB. Even if it has an impact on FPRB, it is often through the channel of ECA. Therefore, the tool variables selected in this paper are reasonable.

In this paper, 2SLS model is used to test the weak instrumental variables. The first-stage regression results show that the impact of instrumental variables on farmers’ ECA is significantly negative at the statistical level of 1%, and the *f*-value of joint significance test is greater than 10, indicating that there is no problem of weak instrumental variables. The test values of Wald and Corr in Table 6 show that there are endogenous problems in the influence of e-commerce on FPRB. After introducing instrumental variables, ECA still has a significant negative impact on farmers’ pesticide application behavior, indicating that ECA can significantly reduce pesticide application and promote pesticide reduction.

**TABLE 6:** Two-stage lease squares (2SLS) and ERM regression results.

Variable name	2SLS		ERM	
	First stage regression	Second stage regression	First stage regression	Second stage regression
	ECA	FPRB	ECA	FPRB
ECA		−5.353*** (1.115)		−1.672*** (0.393)
The spherical distance from Hangzhou to each city	−0.044*** (0.005)		−0.180*** (0.026)	
Control variables	Yes	Yes	Yes	Yes
Constant term	0.148 (0.119)	1.472 (1.051)	−1.714** (0.701)	2.982*** (0.815)
*F*-value	15.55			145.84***
Wald test value	31.35***			
Corr				0.246*** (0.075)
N	3531	3531	3531	3531

**,

***Means significant at the statistical level of 5% and 1% respectively.

### 5.4 Mechanism test

In order to clarify the influence mechanism of ECA on FPRB, this paper uses the intermediary effect method to test the mechanism, and the results are shown in Table 7. The regression results show that ECA can promote farmers to reduce pesticide use by improving agricultural income expectation, food safety perception and promoting agricultural machinery socialization service adoption, and the research hypotheses H2, H3 and H4 are verified.

**TABLE 7:** Test results of mechanism.

Variable name	(1)	(2)	(3)	(4)	(5)	(6)	(7)
	FPRB	Agricultural income expectations	FPRB	Food safety perception	FPRB	Agricultural machinery socialization service adoption	FPRB
ECA	1.236*** (0.117)	0.393*** (0.083)	1.197*** (0.117)	0.496** (0.108)	1.157*** (0.117)	0.823*** (0.074)	1.207*** (0.118)
Agricultural income expectations			0.323*** (0.051)				
Food safety perception					0.760*** (0.067)		
Agricultural machinery socialization service adoption							0.130** (0.062)
Control variables	Yes	Yes	Yes	Yes	Yes	Yes	Yes
R^2^	0.091	0.013	0.100	0.083	0.121	0.093	0.092
N	3531	3531	3531	3531	3531	3531	3531

**,

***Means significant at the statistical level of 5% and 1% respectively.

### 5.5 Heterogeneity test

Due to the difference of individual endowment, family endowment and regional endowment, the resource constraints faced by FPRB are different, which may lead to the heterogeneity of the influence of e-commerce on FPRB in different groups and regions. Therefore, this paper investigates the heterogeneous influence of ECA on FPRB from individual, family and regional levels.

#### 5.5.1 Heterogeneity at the individual level

Referring to the research of Tian et al. (52), in this paper, farmers over 65 years old are defined as elderly farmers, and those under 65 years old are defined as non-elderly farmers. With junior high school education as the dividing line, those who have received education above junior high school are defined as having a high educational attainment, and those who have received education below junior high school are defined as having a low educational attainment, and investigates the differences in the influence of ECA on FPRB under different ages and education levels. From the regression results in Table 8, it can be seen that the influence of ECA on FPRB is heterogeneous in age and education level. From the perspective of age heterogeneity, the pesticide reduction effect of ECA is more obvious for elderly farmers. The reason is that under the influence of urbanization, a large number of young and middle-aged laborers flow to cities, and most of them are elderly farmers engaged in agricultural production in rural areas. Under the influence of rural e-commerce, this group is more likely to reduce pesticide application. Judging from the heterogeneity of education level, compared with farmers with high education level, the pesticide reduction effect of ECA of farmers with low education level is more obvious. The possible reason is that farmers with high education level have more employment choices, prefer to go out to work rather than engage in agricultural production and management, and farmers with low education level often face greater economic pressure. Increasing income is their main behavioral motivation. Reducing the use of pesticides can not only reduce production costs, but also obtain the premium of sustainable products brought by e-commerce.

**TABLE 8:** Heterogeneity test results at individual level.

Variable name	Age		Degree of education	
	Elderly farmers	Non-elderly farmers	Low education level	High education level
ECA	1.407*** (0.194)	1.139*** (0.147)	1.393*** (0.133)	0.924*** (0.247)
Control variables	Yes	Yes	Yes	Yes
Constant term	3.791*** (0.982)	2.614*** (0.578)	1.732*** (0.523)	4.777*** (1.487)
Pseudo R^2^	0.093	0.093	0.086	0.060
N	2407	1124	3047	484

***Means significant at the statistical level of 1%.

#### 5.5.2 Heterogeneity at the family level

This paper explores the heterogeneous influence of ECA on FPRB under different family political identities and family cultivated land scale. According to whether there is party member in the family, the family political identity is regressed in groups, and the family cultivated land scale is regressed in groups with one hectare as the dividing line. From the regression results in Table 9, it can be seen that the influence of ECA on FPRB is heterogeneous in family political identity and family cultivated land scale. Judging from the heterogeneity of family political identity, the pesticide reduction effect of ECA is more obvious for farmers with party member at home. The possible reason is that party member usually has a strong sense of social responsibility and environmental protection, and it is more likely to actively respond to the national policy call for pesticide reduction, and publicize the concept of environmental protection and sustainable production to family members, and guide family members to practice pesticide reduction in agricultural production. Judging from the heterogeneity of family cultivated land scale, compared with farmers with small cultivated land scale, farmers with large cultivated land scale have more obvious pesticide reduction effect. The possible reason is that farmers with large cultivated land often have stronger ability to adopt advanced technologies, such as drone spraying and physical control. This kind of advanced technology can not only directly improve the efficiency of pesticide application, but also fundamentally reduce the probability of pests and diseases by optimizing the production environment, thus reducing the dosage of pesticides.

**TABLE 9:** Heterogeneity test results at family level.

Variable name	Family political identity		Family cultivated land scale	
	Party members	Non-party member	Small scale land	Large scale land
ECA	1.278*** (0.272)	1.148*** (0.135)	1.194*** (0.138)	1.346*** (0.222)
Control variables	Yes	Yes	Yes	Yes
Constant term	5.194*** (1.064)	1.945*** (0.542)	2.749*** (0.537)	4.060*** (0.975)
Pseudo R^2^	0.085	0.089	0.089	0.103
N	796	2735	2681	850

***Means significant at the statistical level of 1%.

#### 5.5.3 Heterogeneity at the regional level

This paper explores the heterogeneous influence of ECA on FPRB under different agricultural functional divisions and geographical locations. Based on whether the province where the farmers are located is a major grain-producing area, the agricultural functional zoning is grouped and returned, and the geographical location is grouped and returned with the Hu Huanyong Line as the dividing line. According to the regression results in Table 10, the influence of ECA on FPRB is heterogeneous in agricultural function zoning and geographical location. Judging from the heterogeneity of agricultural function zoning, the pesticide reduction effect of farmers participating in ECA in major grain producing areas is more obvious. The possible reasons are that agricultural production activities in major grain producing areas are more concentrated, pesticide reduction technology is more popular, farmers are more likely to obtain premium of high-quality agricultural products from e-commerce, and their willingness to reduce pesticides is higher. From the perspective of geographical heterogeneity, compared with farmers located in the west of Hu Huanyong Line, farmers located in the east of Hu Huanyong Line have more obvious pesticide reduction effect when they participate in ECA. The possible reason is that the economic development level in the area east of Hu Huanyong Line is higher, and consumers’ demand for sustainable agricultural products is stronger. Farmers are more likely to improve the quality of products through scientific application of pesticides and reducing the application amount of pesticides, and then obtain high income.

**TABLE 10:** Heterogeneity test results at regional level.

Variable name	Agricultural functional zoning		Geographical location	
	Major grain producing areas	Non-major grain producing areas	East of Hu Huanyong line	West of Hu Huanyong line
ECA	1.622*** (0.195)	1.127*** (0.152)	1.384*** (0.127)	0.566* (0.325)
Control variables	Yes	Yes	Yes	Yes
Constant term	−1.593** (0.653)	10.186*** (0.851)	1.741*** (0.495)	5.510* (2.500)
Pseudo R^2^	0.119	0.171	0.117	0.117
N	1725	1806	2772	759

*,

** and

***Means significant at the statistical level of 10%, 5% and 1% respectively.

### 5.6 Further analysis

#### 5.6.1 Differences in the impact of different e-commerce scales

Influenced by resource endowment, technology application ability and market orientation, the influence of different e-commerce scale on FPRB may be different. Based on the average sales of e-commerce, this paper divides the scale of e-commerce of farmers into scale e-commerce and non-scale e-commerce, and makes regression analysis. According to the regression results in Table 11, compared with non-scale e-commerce, the pesticide reduction effect of scale e-commerce is more obvious. The possible reason is that scale e-commerce operators usually have stronger resource allocation ability and capital advantage, and can adopt more sustainable production technologies and equipment to achieve pesticide reduction. At the same time, scale e-commerce operators have stronger bargaining power and brand influence in the market, and can obtain positive incentives for pesticide reduction through the “high quality and good price” mechanism.

**TABLE 11:** Differences in the impact of different ECA scales.

Variable name	(1)	(2)
Scale e-commerce	0.451** (0.203)	
Non-scale e-commerce		0.005 (0.457)
Control variables	Yes	Yes
Constant term	2.654*** (0.454)	2.601*** (0.645)
Pseudo R^2^	0.056	0.054
N	3531	3531

**,

***Means significant at the statistical level of 5% and 1% respectively.

#### 5.6.2 Differences in the impact of different e-commerce models

With the booming of e-commerce industry, rural e-commerce presents a diversified development trend. At present, rural e-commerce mainly includes two modes: platform e-commerce and social e-commerce (53). Platform e-commerce mode mainly means that farmers set up online stores by entering JD.COM, Taobao, Pinduoduo and other well-known large-scale e-commerce platforms, and use the traffic and resources of these platforms to push agricultural products to a broader market (54). The social e-commerce mode mainly refers to farmers using the social attributes and communication advantages of social platforms such as WeChat, Xiaohongshu and Tik Tok to directly promote and sell agricultural products to consumers.

Considering the heterogeneity of operating rules and market response mechanism of different e-commerce models, this paper further explores the differences in the influence of different e-commerce models on FPRB. According to the regression results in Table 12, compared with the platform e-commerce model, the pesticide reduction effect of social e-commerce model is more obvious. The possible reason is that compared with platform e-commerce, which only provides an online trading platform for consumers and businesses to trade directly, social e-commerce focuses more on using social networks to promote and sell goods. With the help of social media platform, farmers recommend products to potential consumers through user sharing, likes and comments, and realize word-of-mouth marketing and spontaneous communication of users. In order to ensure product quality and reputation, farmers will pay more attention to the quality of agricultural products, thus reducing the use of pesticides.

**TABLE 12:** Differences in the impact of different e-commerce models.

Variable name	(1)	(2)
Platform e-commerce	0.248 (0.490)	
Social e-commerce		0.401** (0.160)
Control variables	Yes	Yes
Constant term	2.602*** (0.453)	2.631*** (0.454)
Pseudo R^2^	0.054	0.056
N	3531	3531

**,

***Means significant at the statistical level of 5% and 1% respectively.

#### 5.6.3 Difference in the impact of different pesticide application methods

At present, pesticide application methods in China are mainly divided into mechanical pesticide application and manual pesticide application. In terms of mechanical pesticide application, new types of plant protection machinery such as agricultural drones and self-propelled boom sprayers are gradually becoming popular. They have high operational efficiency, high pesticide utilization rate, and can effectively reduce pesticide drift and environmental pollution. However, the promotion of these new machines still faces the cost and technical threshold, and it is difficult for some small farmers to widely apply them. In the aspect of manual application of pesticides, small manual sprayers still dominate, but this method has high labor intensity, low efficiency and low effective deposition rate of pesticides, which is easy to cause problems such as excessive pesticide residues and environmental pollution.

The emergence of e-commerce provides a new path for optimizing agricultural pesticide application methods. The information transparency mechanism and convenience of e-commerce platform can help farmers to obtain new plant protection machinery and sustainable pesticide information more directly, reduce procurement costs, and provide accurate guidance for the promotion of pesticide reduction technology through data analysis and user feedback mechanism. Therefore, this paper further discusses the influence of ECA on pesticide application methods. From the regression results in Table 13, it can be seen that ECA has promoted farmers to apply pesticides mechanically. The possible reason is that e-commerce helps farmers to grasp the market demand more accurately. In order to enhance the market competitiveness, farmers tend to adopt more scientific and reasonable mechanical application methods to reduce labor input costs, improve the uniformity and accuracy of application, and thus ensure the stability and high quality of crop output. This result also confirms the pesticide reduction effect of ECA from the side.

**TABLE 13:** Differences in the impact of different pesticide application methods.

Variable name	Mechanical pesticide application	Manual pesticide application
ECA	0.195** (0.075)	−0.186** (0.075)
Control variables	Yes	Yes
Constant term	−2.587*** (0.444)	2.489*** (0.441)
Pseudo R2	0.027	0.025
N	3531	3531

**,

***Means significant at the statistical level of 5% and 1% respectively.

## 6 Discussion

Under the background of digital and sustainable transformation of rural industries, based on the survey data of 3531 farmers in 10 provinces of China, this paper empirically analyzes the influence of ECA on FPRB. Firstly, The results show that ECA has a positive effect on FPRB, which ECA is consistent with the research conclusion of Wang et al. (30) and (55) confirming that ECA has a positive effect on pesticide reduction. Besides, Yan et al. (56) also reached a similar conclusion, proving that digital economy participation promotes agricultural green production. But this paper is different from it in that pesticide reduction in agricultural green production is discussed separately. The reason is that pesticides have dual attributes in agricultural production, and consumers are highly sensitive to pesticide residues, which are regarded as important indicators for judging food safety. Therefore, refining the research perspective from green production to pesticide reduction is conducive to establishing clearer causal relationship and deeper mechanism analysis, so as to put forward more clear policy recommendations.

When discussing the impact mechanism, this paper uses agricultural income expectations, food safety perception and agricultural machinery socialization services adoption as intermediary variables to clarify the impact mechanism of ECA on pesticide reduction from multiple angles. This is similar to a number of articles that explore price incentives and information acquisition mechanisms (57). However, unlike most articles that directly discuss socialization services and pesticide reduction (47, 58–60), this paper takes agricultural machinery socialization services as an intermediary variable, which not only further verifies the role of socialization services in pesticide reduction, but also builds a bridge between e-commerce management and socialization services.

In addition, this paper further discusses the influence of different e-commerce scales and operation models on FPRB and the influence of ECA on different pesticide application methods, which enriches the research content of ECA and pesticide reduction. When discussing different ECA models, this paper finds that social commerce has more obvious pesticide reduction effect, which is contrary to the conclusion of Li et al. (57). The reasons may be the differences of sample size, geographical location, investigation time and agricultural products investigated. Li et al. (57) investigates vegetable cultivation, and platform e-commerce usually handles bulk vegetables, relies on standardized supply chain, pesticide reduction may be achieved through collective certification. While social e-commerce may involve more small farmers or specialty agricultural products, in which producers interact directly with consumers and pesticide reductions are easier to monitor and verify.

The theory of sustainable development holds that it is necessary to balance economy and ecology effectively, taking into account the dual development goals of economic growth and ecological benefits. The incentive compatibility of economic and ecological goals depends on the application of new technologies and new development models. E-commerce, as a typical representative of the new development model, the integration of resource elements brought by its embedded digital technology not only promotes economic growth, but also promotes the transformation of agricultural production mode, which has positive environmental effects. The theoretical contribution of this paper is to explore the role of digital economy model represented by e-commerce in the sustainable transformation of farmers’ production mode from the micro-behavior of farmers, which has important reference value for promoting the sustainable transformation of small farmers in developing countries.

## 7 Conclusions and policy recommendations

### 7.1 Conclusion

Based on the micro-survey data of 3531 farmers in 10 provinces of China, this paper empirically analyzes the influence of ECA on FPRB and the mechanism of agricultural income expectations, food safety perception and adoption of agricultural machinery socialization service by using binary Logit model and intermediary effect model. Through the above analysis, the following four conclusions can be drawn.

(1)    ECA has significantly promoted the reduction of pesticides for farmers. ECA encourages farmers to reduce pesticide application by breaking information asymmetry, constructing market mechanism of high quality and good price, and providing support for pesticide reduction technology.
(2)    Agricultural income expectation, food safety perception and agricultural machinery socialization service adoption play a positive mediating role in the influence of ECA on FPRB. ECA promotes farmers’ pesticide reduction by raising agricultural income expectation, enhancing food safety perception and promoting the adoption of socialized agricultural machinery services.
(3)    The pesticide reduction effect of ECA varies with individual endowment, family endowment and regional endowment of farmers. Specifically, the pesticide reduction effect of ECA is more obvious for elderly farmers, farmers with low education level, farmers with party members and large scale land in their families, farmers in major grain producing areas and farmers east of Hu Huan Yong Line.
(4)    Compared with non-scale e-commerce and platform e-commerce, the pesticide reduction effect of scale e-commerce and social e-commerce is more obvious. Compared with manual application, e-commerce operation can promote farmers to adopt mechanical application mode.

### 7.2 Policy implications

In the era of digital economy, ECA is an important way to promote pesticide reduction and sustainable transformation of agriculture. Based on the above research conclusions, this paper draws the following four policy implications.

(1) Multi-subjects should work together to effectively promote farmers to integrate into the e-commerce market. The government should strengthen information logistics infrastructure to realize full coverage of rural network and express delivery; agricultural technology departments should train farmers’ e-commerce skills through online teaching and field guidance to improve operation ability; e-commerce organizations should give full play to channel and information advantages to guide farmers to reduce pesticide use and assist green transformation of agriculture.

(2) The government should improve agricultural price support mechanisms and subsidize farm machinery services to stabilize farmer income and modernize production. Agricultural departments must enhance food safety education and establish a full-chain traceability system to raise safety awareness. E-commerce platforms should be encouraged to participate in rural e-commerce, expanding sales channels and providing market support for safe production.

(3) The government should implement differentiated e-commerce support policies to narrow the urban-rural and inter-group digital divide and ensure equitable sharing of digital benefits. It needs to enhance digital skills training for vulnerable farmers and allocate more resources to non-grain producing areas and regions west of the Hu Huanyong Line. These efforts will integrate e-commerce into agricultural production and support green agricultural development.

(4) Local governments should expand e-commerce scale based on local conditions and leverage its role in reducing pesticide use and increasing efficiency. They should enhance farmer training to improve social commerce operational skills and foster business model innovation. Additionally, mechanization of pesticide application should be accelerated to enhance precision and efficiency, reduce overuse, and support green, high-quality agricultural development.

## Data Availability

Publicly available datasets were analyzed in this study. This data can be found here: http://rdi.cssn.cn/dcsj/202306/t20230607_5643271.shtml.
